# Novel Nonreplicating Vaccinia Virus Vector Enhances Expression of Heterologous Genes and Suppresses Synthesis of Endogenous Viral Proteins

**DOI:** 10.1128/mBio.00790-17

**Published:** 2017-06-06

**Authors:** Linda S. Wyatt, Wei Xiao, Jeffrey L. Americo, Patricia L. Earl, Bernard Moss

**Affiliations:** Laboratory of Viral Diseases, National Institute of Allergy and Infectious Diseases, National Institutes of Health, Bethesda, Maryland, USA; University of Pittsburgh School of Medicine

**Keywords:** bacteriophage T7 RNA polymerase, immune response, influenza virus, live vector vaccines, poxvirus, recombinant virus, vaccinia virus

## Abstract

Viruses are used as expression vectors for protein synthesis, immunology research, vaccines, and therapeutics. Advantages of poxvirus vectors include the accommodation of large amounts of heterologous DNA, the presence of a cytoplasmic site of transcription, and high expression levels. On the other hand, competition of approximately 200 viral genes with the target gene for expression and immune recognition may be disadvantageous. We describe a vaccinia virus (VACV) vector that uses an early promoter to express the bacteriophage T7 RNA polymerase; has the A23R intermediate transcription factor gene deleted, thereby restricting virus replication to complementing cells; and has a heterologous gene regulated by a T7 promoter. In noncomplementing cells, viral early gene expression and DNA replication occurred normally but synthesis of intermediate and late proteins was prevented. Nevertheless, the progeny viral DNA provided templates for abundant expression of heterologous genes regulated by a T7 promoter. Selective expression of the *Escherichia coli lac* repressor gene from an intermediate promoter reduced transcription of the heterologous gene specifically in complementing cells, where large amounts might adversely impact VACV replication. Expression of heterologous proteins mediated by the A23R deletion vector equaled that of a replicating VACV, was higher than that of a nonreplicating modified vaccinia virus Ankara (MVA) vector used for candidate vaccines *in vitro* and *in vivo*, and was similarly immunogenic in mice. Unlike the MVA vector, the A23R deletion vector still expresses numerous early genes that can restrict immunogenicity as demonstrated here by the failure of the prototype vector to induce interferon alpha. By deleting immunomodulatory genes, we anticipate further improvements in the system.

## IMPORTANCE

Vaccines provide an efficient and effective way of preventing infectious diseases. Nevertheless, new and better vaccines are needed. Vaccinia virus, which was used successfully as a live vaccine to eradicate smallpox, has been further attenuated and adapted as a recombinant vector for immunization against other pathogens. However, since the initial description of this vector system, only incremental improvements largely related to safety have been implemented. Here we described novel modifications of the platform that increased expression of the heterologous target gene and decreased expression of endogenous vaccinia virus genes while providing safety by preventing replication of the candidate vaccine except in complementing cells used for vector propagation.

## INTRODUCTION

The expression of heterologous genes by vaccinia virus (VACV) was described more than 30 years ago (1–3), and recombinant vectors have been extensively deployed since then for immunology and infectious disease research (4, 5). Over the years, the system has been improved with stronger promoters (6, 7), newer methods of selecting recombinant viruses (8–11), and safer replication-deficient poxvirus strains (12–14). Numerous veterinary and candidate human vaccines are based on this technology (15). Nevertheless, aside from the incremental advances referred to above, there have been few innovations to the platform itself. One novel modification is the insertion of the bacteriophage T7 DNA-dependent RNA polymerase gene into the VACV genome, allowing selective high expression of genes regulated by the T7 promoter (16, 17). A second innovation is the regulation of the T7 RNA polymerase and promoter with the *Escherichia coli lac* operator and Lac repressor providing an inducible system for high-level *in vitro* expression of recombinant proteins (18, 19). Holzer and coworkers (20) made another innovation by deleting a gene essential for viral DNA replication and propagating the defective virus in a complementing cell line. Although the absence of postreplicative viral gene expression in noncomplementing cells is an important feature of that system, only early promoters, which are relatively weak, could be used for expression of heterologous genes.

Here we describe a novel VACV vector that achieves high expression of heterologous genes by use of the T7 RNA polymerase and promoter while also preventing expression of viral intermediate and late genes because of the deletion of an essential intermediate transcription factor gene. In addition, through use of the *Escherichia coli lac* repressor-operator system, heterologous gene expression is minimized in the complementing cell line used for vector propagation. The new system does not require use of an inducer and fulfills many desirable criteria for a vaccine platform, including (i) the safety of a nonreplicating vector; (ii) high expression of recombinant proteins *in vitro* and *in vivo*; (iii) low expression of endogenous vector proteins; and (iv) stable propagation of the vector with low expression of recombinant proteins in complementing cells.

## RESULTS

### Features of the expression system.

**FIG 1:**
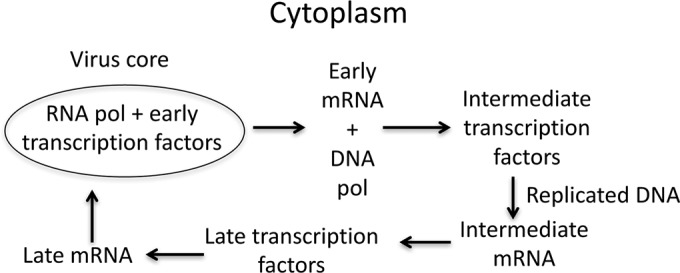
Outline of poxvirus transcription program. RNA polymerase (pol) in association with early transcription factors transcribes the viral genome within the virus core to produce early mRNAs. The latter are translated in the cytoplasm to produce DNA pol, additional replication proteins, and intermediate transcription factors. Newly synthesized DNA serves as a template for transcription by RNA polymerase (pol) and intermediate transcription factors. Intermediate mRNAs encode late transcription factors, and late mRNAs are synthesized. Late mRNAs encode early transcription factors and structural proteins, which are assembled into virus particles for the next round of infection.

**FIG 2:**
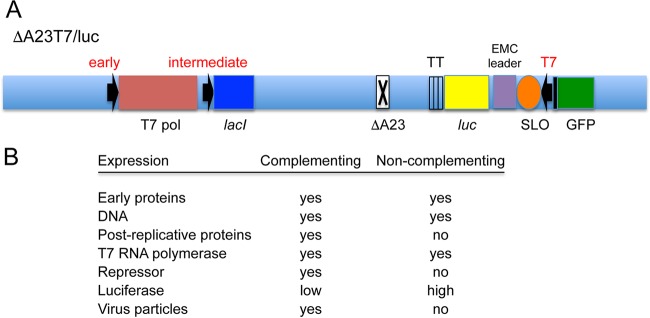
Design and properties of the ΔA23T7 vector system. (A) Features of the recombinant VACV genome of ΔA23T7/luc vector are depicted in the diagram. The early, intermediate, and T7 promoters are indicated in red and placed next to the ORFs encoding the T7 polymerase (T7 pol), *lac* repressor (*lacI*), and *luc*, respectively. Additional abbreviations: X, deleted A23 intermediate transcription factor gene; TT, triple-transcription terminator; SLO, modified *lac* operator; GFP, green fluorescent protein ORF; EMC leader, encephalomyocarditis virus (EMCV) translational enhancer region. (B) Predicted effects on gene expression in complementing and noncomplementing cells.

The new vector platform is based on the manipulation of the poxvirus transcription system, which is outlined in Fig. 1. Poxvirus gene expression occurs in the cytoplasm and is programed into early, intermediate, and late phases by a multisubunit DNA-dependent RNA polymerase and stage-specific transcription factors that recognize cognate promoters (21). The RNA polymerase and protein factors that are packaged with the genome in the core of the infecting virus particle transcribe early genes, whereas *de novo* synthesized viral proteins transcribe the intermediate and late genes of progeny DNA. Programed synthesis is achieved by sequential expression of the intermediate, late, and early transcription factors. Only early genes are expressed if either DNA replication or synthesis of intermediate transcription factors is prevented. Although the two intermediate transcription factors A8 and A23 encoded by the A8R and A23R genes (22) are each essential for virus replication, null mutants can be propagated in a cell line that constitutively expresses these proteins (23). Our new vector (Fig. 2A) is based on a recombinant VACV in which the A23R open reading frame (ORF) was deleted and is therefore capable of expressing genes with only early promoters in noncomplementing cells. However, selective high expression of a heterologous gene regulated by a T7 promoter and encephalomyocarditis virus translational enhancer was achieved by incorporating the T7 RNA polymerase ORF controlled by an early promoter in the VACV genome. Because early gene expression was sufficient for viral DNA replication, thousands of copies of the VACV genome containing the heterologous gene template, which can be transcribed by the T7 RNA polymerase, were made even in noncomplementing cells. Additionally, the *E. coli lac* operator system reduced expression of the heterologous gene in the complementing cell line to minimize potential adverse effects of overexpression on virus replication. The latter was achieved by using an intermediate promoter to regulate the *E. coli* Lac repressor and by placing the *lac* operator adjacent to the T7 promoter. The modified genome of the new VACV vector designed to express firefly luciferase (Luc) is shown in Fig. 2A, and the predicted effects on gene expression in complementing and noncomplementing cells are outlined in Fig. 2B. The new vector was named ΔA23T7 to indicate the absence of the intermediate transcription factor and the presence of the T7 transcription system. For brevity, the presence of the *E. coli lac* operator system was not included in the name. Table 1 lists the recombinant viruses used in this study and their chief characteristics for easy reference.

**TABLE 1:** Recombinant viruses

Recombinant virus	Parent	A23R	T7 pol	*lacI*	Promoter/target
ΔA23	WR^a^	−	−	−	None
ΔA23T7	WR	−	+	+	T7
ΔA23T7/luc^b^	WR	−	+	+	T7/luc
ΔA23T7/HA^c^	WR	−	+	+	T7/HA
WRvFIRE	WR	+	−	−	Syn EL^d^/luc
T7/luc	WR	+	+	+	T7/luc
T7LacOI	WR	+	+	+	None
MVA/luc	MVA^e^	+	−	−	Syn EL/luc
MVA/HA	MVA	+	−	−	mH5^f^/HA
WR/HA	WR	+	−	−	7.5^g^/HA

a WR strain of VACV.

b Firefly luciferase.

c Influenza virus hemagglutinin.

d Strong synthetic early-late promoter.

e Modified vaccinia virus Ankara.

f Modified H5 promoter.

g 7.5 promoter.

### Conditional replication of ΔA23T7/luc.

**FIG 3:**
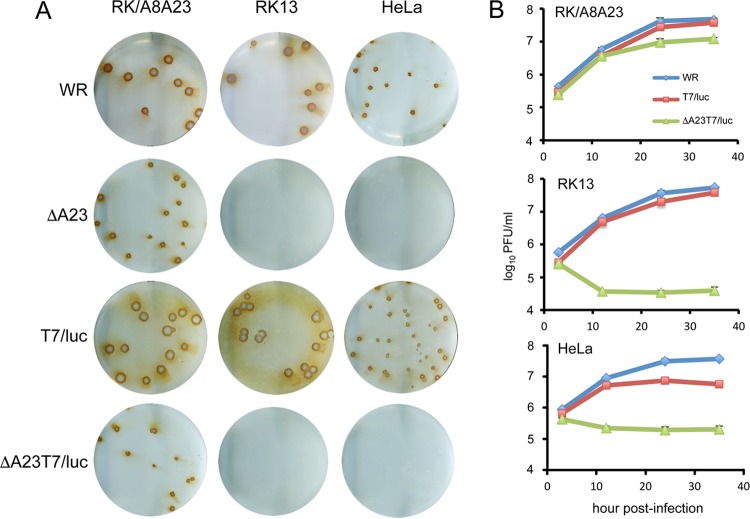
Conditional replication of recombinant vector. (A) Plaque formation. RK/A8A23, RK13, and HeLa cells were infected with parental VACV strain WR (WR) and T7/luc as replication-competent controls and VACV strain WR with deletion of A23 ORF (ΔA23) and ΔA23T7/luc and incubated for 3 days at 37°C. Plaques were stained with VACV antibody and protein A conjugated to peroxidase. (B) Virus yields. The cell lines described above were infected with 5 PFU per cell of the indicated viruses and harvested at 2, 12, 24, and 36 h. The cells were then lysed and virus titers determined by plaque assay in RK/A8A23 cells.

The parental VACV Western Reserve (WR) strain as well as T7/luc, a replication-competent virus expressing T7 RNA polymerase and the Lac repressor, formed plaques in HeLa, RK13, and RK/A8A23 cells, whereas ΔA23T7/luc, like the original ΔA23 virus, formed plaques only in complementing RK/A8A23 cells (Fig. 3A). A corresponding result was obtained by determining virus yields: the ΔA23T7/luc mutant produced infectious virus only in RK/A8A23 cells (Fig. 3B).

**FIG 4:**
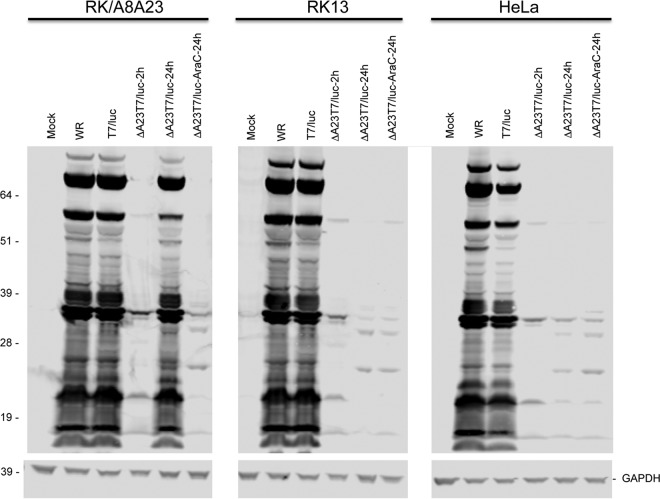
Viral protein synthesis in complementing and noncomplementing cells. RK/A8A23, RK13, and HeLa cells were infected with 5 PFU/cell of VACV strain WR (WR) or T7/luc for 24 h or with 5 PFU/cell of ΔA23T7/luc vector for 2 or 24 h in the presence or absence of AraC. The cells were lysed, the proteins resolved by SDS polyacrylamide gel electrophoresis, and the viral proteins detected by immunoblotting with antiserum to VACV. GAPDH was detected with a specific antibody as a gel loading control. The masses (in kilodaltons) and positions of marker proteins are indicated on the left.

The replication defect of ΔA23T7/luc in noncomplementing cells was associated with a profound block in viral protein synthesis. Early viral proteins are mainly involved in transcription, replication, and immune defense and are expressed at relatively low levels, whereas intermediate and late gene products include the abundant proteins forming the virus particle. The major bands corresponding to viral intermediate and late proteins were detected in RK/A8A23, RK13, and HeLa cells at 24 h after infection with control strain VACV WR and T7/luc as shown by immunoblotting with VACV antiserum (Fig. 4). In contrast, the major proteins were detected in the complementing cells infected with the ΔA23T7/luc mutant at 24 h but not in either RK-13 or HeLa cells. Faint bands, corresponding to proteins made at 2 h after infection and in the presence of the DNA synthesis inhibitor AraC, were detected in extracts from HeLa and RK13 infected for 24 h with ΔA23T7/luc.

### Expression of LacI and Luc in cultured cells.

**FIG 5:**
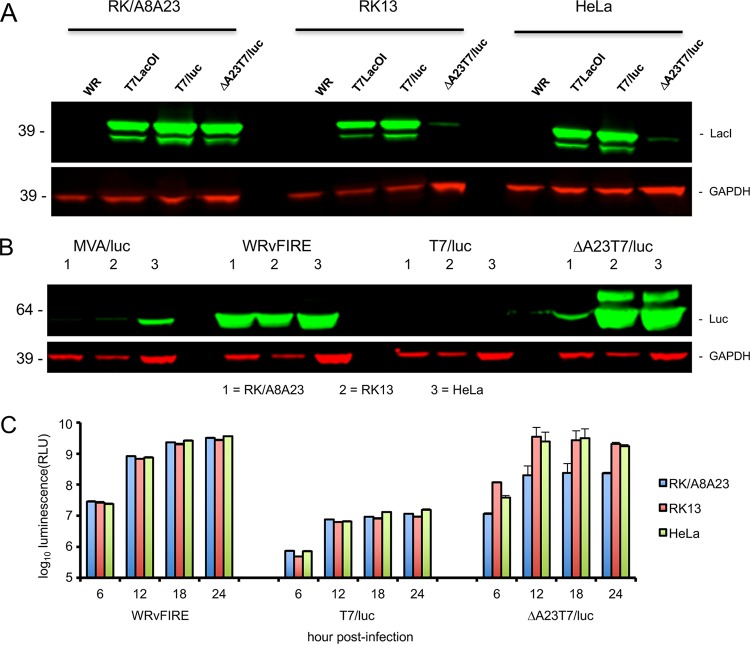
LacI and Luc synthesis. (A) RK/A8A23, RK13, and HeLa cells were infected with VACV WR (WR), T7LacOI, or T7/luc replication-competent virus or with replication-defective ΔA23T7/luc virus at a multiplicity of 5 PFU/cell. After 24 h, the cells were lysed and LacI was detected by SDS polyacrylamide gel electrophoresis followed by immunoblotting and probing with specific antibody. Antibody to GAPDH was used as a loading control. (B) The experimental procedure was like that described for panel A except that the blot was probed with antibody to Luc. (C) Luc activity was detected by luminescence at 6, 12, 18, and 24 h after infection of cells as described for panels A and B, and the data were plotted on a log scale. RLU, relative light units.

Based on the vector design (Fig. 2), ΔA23T7/luc should express Lac repressor only in complementing RK/A8A23 cells, which synthesize the intermediate transcription factors. For positive controls, we determined LacI synthesis in cells infected with replication-competent viruses T7LacOI and T7/luc that retain the A23 gene. Those control viruses made similar amounts of LacI in RK/A8A23, RK13, and HeLa cells (Fig. 5A). In contrast, when the three cell lines were infected with ΔA23T7/luc, a strong LacI band was present only in lysates of RK/A8A23 cells; the trace bands in RK13 and HeLa cells may have represented residual LacI from the RK/A8A23 cells in which the virus was propagated.

A second prediction was that more Luc should be made by ΔA23T7/luc in noncomplementing RK13 and HeLa cells than in complementing RK/A8A23 cells (Fig. 2B). For controls, we used the replication-competent WRvFire and the replication-deficient modified vaccinia virus Ankara (MVA)/luc, both of which express Luc from the strong synthetic early/late promoter (6). As predicted, ΔA23T7/luc expressed larger amounts of Luc in HeLa and RK13 cells than in RKA8A23 cells because repressor is made in the latter (Fig. 5B). In contrast, the level of Luc synthesis in all cells infected with T7/luc, which retains the A23 gene and therefore expresses the repressor in each cell line, was too low to detect by immunoblotting (Fig. 5B). Because of the specific inhibition of viral late protein synthesis in rabbit (but not human or mouse) cells due to the absence of the K1 gene (24), expression of Luc by the MVA construct was low in the RK13 and RKA8A23 cells but was also lower than that seen with ΔA23T7/luc in HeLa cells (Fig. 5B).

Measurement of Luc activity was more sensitive and quantitative than immunoblotting and, for example, allowed detection of the low expression by T7/luc (Fig. 5C). In the three cell lines infected with ΔA23T7/luc, activity was detected at 6 h and plateaued at 12 h. However, the activity detected in the noncomplementing cells was over 1 log higher than in the RKA8A23 cells in which the repressor was synthesized. Luc activity in HeLa and RK13 cells infected with WRvFire increased more slowly than with ΔA23T7/luc, although the final values were similar. Thus, the expression of the reporter gene by ΔA23T7/luc in noncomplementing cells was equivalent to that achieved using one of the strongest known promoters in a replicating vector.

### *In vivo* synthesis of Luc.

**FIG 6:**
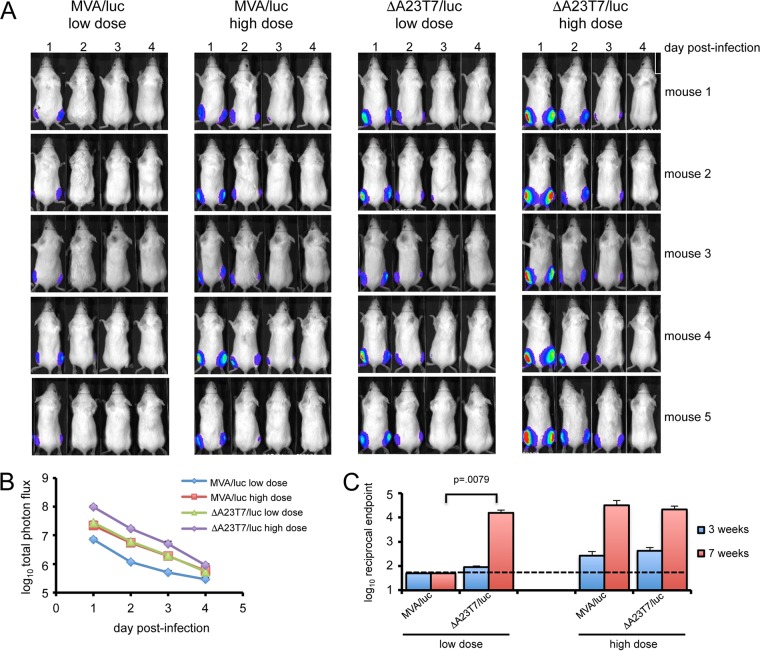
Luc synthesis in mice by MVA and ΔA23T7 vectors. (A) Mice (*n* = 5) were injected intramuscularly with 5 × 10^5^ (low dose) or 5 × 10^6^ (high dose) PFU of MVA/luc or ΔA23T7/luc. Luciferin was injected intraperitoneally at daily intervals and luminescence determined by whole-animal imaging. (B) The total photon flux on each day was averaged for the 5 animals in each group shown in panel A and the data plotted on a log scale. (C) The mice were boosted with a second inoculation of MVA/luc or ΔA23T7/luc at 4 weeks, and Luc antibody titers were determined by ELISA on blood obtained at 3 weeks after the first and second inoculations. Data are displayed on a log scale, and the dashed line indicates the limit of detection of the assay.

Live-animal imaging was used to detect Luc synthesis in mice infected intramuscularly (i.m.) with 5 × 10^5^ or 5 × 10^6^ PFU of ΔA23T7/luc or MVA/luc. The same settings were used throughout so that the image intensities can be compared directly in Fig. 6A. Because MVA and the A23R deletion mutant are replication defective in mice, luminescence was largely restricted to the site of inoculation and was maximal on day 1 and gradually decreased thereafter. The luminescence intensity was greater with the high dose than with the low dose of each virus (*P* < 0.001) and higher with ΔA23T7/luc than with MVA/luc (*P* < 0.001 on days 1 to 3 for both doses) (Fig. 6B).

The animals from the experiment described above were bled to determine the Luc antibody (Ab) titers. At 3 weeks after the low-dose (5 × 10^5^ PFU) virus inoculations, the antibody titers were low for the animals receiving either MVA/luc or ΔA23T7/luc (Fig. 6C). A second virus inoculation was given at 4 weeks to determine if the antibody titers could be boosted. The antibody level increased more than 100-fold (*P* < 0.008) after the second low dose of ΔA23T7/luc but remained low after the second low dose of MVA/luc (Fig. 6C). In contrast, boosting increased the Luc antibody titers to similar levels after the high-dose (5 × 10^6^ PFU) inoculations of both viruses (Fig. 6C). Thus, synthesis of Luc was higher in mice infected with 5 × 10^5^ or 5 × 10^6^ PFU of ΔA23T7/luc than MVA/luc and at low inoculation doses induced more Luc antibody.

### Recombinant vectors expressing influenza virus hemagglutinin (HA).

**FIG 7:**
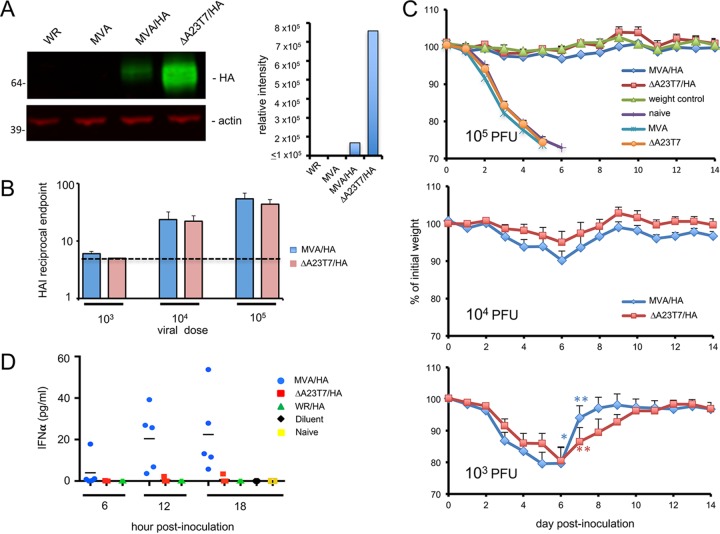
Synthesis, immunogenicity, and protective efficacy of influenza virus HA expressed by MVA and ΔA23T7 vectors. (A) *In vitro* synthesis of HA. HeLa cells were infected with 3 PFU/cell of VACV WR (WR), MVA, MVA/HA, and ΔA23T7/HA viruses. After 24 h, the cells were harvested and lysates analyzed by SDS polyacrylamide gel electrophoresis and immunoblotting with a MAb to HA and to actin followed by IRDye secondary antibodies. The relative intensity of antibody staining was determined with a Li-COR imager. (B) HA antibody production. Mice were inoculated i.m. with a single dose of 10^3^, 10^4^, or 10^5^ PFU of MVA or ΔA23T7 vector or with 10^5^ PFU of MVA/HA or ΔA23T7 virus. After 3 weeks, hemagglutination inhibition (HAI) antibody titers were determined in sera of individual mice. The titers given are averages from results of two independent experiments. The data are plotted on a log scale, and the dashed line indicates the limit of detection of the assay. (C) Influenza virus challenge. At 4 weeks after i.m. immunization with 10^3^ (bottom panel), 10^4^ (middle panel), or 10^5^ (top panel) PFU of MVA/HA or ΔA23T7/HA or with 10^5^ PFU of MVA or ΔA23T7 viruses, the mice were challenged intranasally with a lethal dose of influenza virus A-PR8/34. Weights were determined daily. *, the day a mouse died or was sacrificed due to severe weight loss. (D) IFN-α induction. Groups of mice (*n* = 5) were inoculated intravenously with 10^6^ PFU of MVA/HA, ΔA23T7/HA, or WR/HA virus. At 6, 12, and 18 h, the mice were bled and sacrificed. Additional mice were left uninfected (naive) and bled at the 18-h time point. IFN-α levels were measured in plasma from individual mice using an ELISA-based assay. The assay was repeated and the average plotted. The bars represent the mean values for mice in each group.

Another recombinant virus, ΔA23T7/HA, containing the influenza HA ORF from the A-PR8/34 strain of influenza virus instead of the *luc* ORF, was constructed. For comparison, the same HA ORF regulated by the moderately strong early/late mH5 promoter frequently used for candidate vaccines (25) was inserted into MVA. The expression level, determined by immunoblotting with antibody to HA, was 4.5- to 7-fold higher in HeLa cells infected with ΔA23T7/HA than in those infected with MVA/HA (Fig. 7A and data not shown). The HA antibody responses elicited by i.m. inoculation of 10^3^, 10^4^, or 10^5^ PFU of ΔA23T7HA or MVA/HA were determined 3 weeks later. Mice were also inoculated with 10^5^ PFU of MVA or ΔA23T7, neither of which expressed HA, as negative controls. The HA antibody titers from mice infected with ΔA23T7/HA or MVA/HA were elevated at doses of 10^4^ and 10^5^ PFU and were similar for each virus (Fig. 7B). Both viruses also induced VACV antibodies that increased with the size of the inoculum (data not shown). Upon intranasal challenge with 100 50% lethal doses (LD_50_) of influenza virus, the control mice succumbed within a week, whereas the survival rates were 3/5, 5/5, and 5/5 for mice immunized with 10^3^, 10^4^, and 10^5^ PFU of ΔA23T7/HA and 2/5, 5/5, and 5/5 for mice immunized with 10^3^, 10^4^, and 10^5^ PFU MVA/HA. Thus, the levels of immunogenicity and protective efficacy of ΔA23T7/HA and MVA/HA were similar despite the differences in expression *in vitro*.

### Differences in induction of interferon alpha (IFN-α) by ΔA23T7/HA and MVA/HA.

MVA was generated by serial passaging of the parental strain in chicken embryo fibroblasts, during which it lost many genes, including those encoding immunomodulators (26). The loss of such genes is thought to enhance the immunogenicity of MVA (27, 28). Indeed, intravenous inoculation of C57BL/6 mice with MVA was previously shown to induce IFN-α, which potentiates the immune response, but this did not occur when VACV WR (the parent of the ΔA23T7 mutant) was inoculated (29, 30). We carried out a similar experiment to compare the level of induction of IFN-α by ΔA23T7/HA to that by MVA/HA. BALB/c mice were inoculated intravenously with 10^6^ PFU of MVA/HA, VACV WR/HA, or ΔA23T7/HA. At 6, 12, and 18 h, the mice were bled and the amounts of IFN-α determined. Naive (uninfected) mice were also bled at the 18-h time point. IFN-α was detected at 6 h, and the level increased at 12 and 18 h in the mice infected with MVA/HA, but no IFN-α was detected in naive mice or mice infected with VACV WR/HA, and the amounts in animals infected with ΔA23T7/HA were barely detectable (Fig. 7D).

## DISCUSSION

VACV and other poxviruses have several advantages as expression vectors, including the ability to incorporate 25,000 bp or more of heterologous DNA (31), the presence of a cytoplasmic site of transcription, relatively high expression levels, and the ability to infect a wide range of cells and animals. On the other hand, the presence of approximately 200 viral genes, which could compete with the target gene for expression and immune recognition, represents a potential disadvantage. To overcome this drawback, we deleted the gene encoding an intermediate transcription factor so that neither intermediate nor late genes could be expressed but, importantly, early gene expression and viral DNA replication still occurred. To take advantage of the thousands of progeny DNA templates for recombinant gene expression, we inserted a T7 promoter and encephalomyocarditis virus translational enhancer controlling the target gene and incorporated the T7 RNA polymerase gene with an early promoter. Small amounts of T7 RNA polymerase were sufficient to drive this system because the polymerase is catalytically active and only the recombinant gene has a T7 promoter. Indeed, we chose for this first iteration of the system the extremely weak thymidine kinase promoter to regulate the T7 RNA polymerase gene (32). Stronger early promoters are available, and their ability to increase expression even more will be tested in next-generation vectors. To propagate the defective virus, we used a cell line that encodes both of the intermediate transcription factors, which form a heterodimer (23). On the basis of earlier studies, we were concerned that high expression of the recombinant protein might adversely affect virus replication and lead to the selection of nonexpressing mutants during the preparation of large virus stocks (16, 33). To minimize expression of the target protein in complementing cells, we incorporated the gene encoding the *E. coli lac* repressor regulated by an intermediate promoter in the recombinant virus and placed a *lac* operator next to the target gene. Importantly, because the repressor is made only in the complementing cells, expression of the target gene is not reduced in noncomplementing cells. Our studies confirmed the high expression of the target gene and the absence of intermediate and late gene expression in noncomplementing cells. Furthermore, the new vector expressed a *luc* reporter gene at the same level as a replication-competent VACV that employed one of the strongest early/late promoters.

Poxvirus vectors that exhibit low or no replication in human and some other mammalian cells, including avipoxviruses, NYVAC, and MVA, have shown promise as safe vaccines, although the precise basis for the host range restriction remains uncertain (34–36). The absence of a gene encoding an essential transcription factor accounts for the inability of ΔA23 vectors to replicate in any noncomplementing cell as well as their localization at the site of intramuscular injection in mice, causing no discernible disease. Studies, particularly those performed with MVA, have indicated two features that are important for immunogenicity. One is the level of target gene expression (37), and the other is the loss of many immune defense genes, which occurred during passages in chicken embryo fibroblasts (26). Unlike other strains of VACV, MVA does not express soluble proteins that bind IFN-α/β, tumor necrosis factor (TNF), IFN-γ, and chemokines (27). Still higher immunogenicity has been obtained by deleting additional immune defense genes from MVA (38–40). In contrast to the MVA vector, the parent WR VACV strain that was the source of the ΔA23T7 viruses retains a large repertoire of immunomodulatory genes. In this context, Waibler and coworkers reported that MVA but not VACV WR induces an IFN-α response in mice (29, 30). We found that MVA/HA induced a robust IFN-α response whereas IFN-α was barely detected in mice infected with ΔA23T7/HA. Numerous studies have shown that type 1 IFNs have an important role in the development of an effective adaptive immune response (41).

Although the high expression of heterologous proteins by ΔA23T7 vectors should favor a strong immune response, the absence of many immunomodulatory genes might give MVA an advantage. In the present study, we found that at a low inoculation dose, the prototype ΔA23T7/luc virus induced a higher antibody response to the reporter protein than an MVA construct. However, this difference was mitigated at higher inoculation doses and when influenza HA, a highly immunogenic membrane-associated protein, was expressed. At doses of 10^4^ PFU or higher, both vectors protected all mice from an intranasal influenza virus challenge. In next-generation ΔA23T7 vectors, we will explore the effects of deleting immune defense genes, which are mostly expressed from early promoters, and of increasing the expression of heterologous proteins regulated by the T7 RNA polymerase.

## MATERIALS AND METHODS

### Cells and viruses.

The construction of RK/A8A23 cells and propagation in medium supplemented with 50 µg/ml of zeocin (Thermo Fisher Scientific) was previously described (23). The VACV Western Reserve (WR) strain (ATCC VR-1354) and MVA 1974 NIH clone 1 (42) and recombinant viruses WRvFire (43), T7lacOI (44), and MVA/gfpluc (obtained from Zain Bengali and referred to here as MVA/luc) have previously been described. All viruses were purified by sedimentation through a 36% sucrose cushion, and the pellet was suspended in 1 mM Tris-HCl (45). For animal experiments, the viruses were further purified by sedimentation on a 24% to 40% sucrose gradient and the bands collected. Influenza virus A/PR/8/34 was grown in the allantoic cavity of 10-day-old embryonated chicken eggs and stored at −80°C.

### Construction of transfer plasmids and recombinant viruses.

Plasmid transfer vector pRB21 (11) was used to insert the T7 RNA polymerase and *E. coli lac* repressor ORFs into the VACV strain WR genome. The strong intermediate I1L promoter (sequence, TTTGTATTTGTATTTAAAAGTTGTTTGGTGAACTTAA) was joined to the *lac* repressor ORF by PCR and inserted into the RB21 plasmid. The VACV early TK promoter (sequence, CGAATAAAGTGAACAATAATTAATTCTTTATTGTCATC) was joined to the T7 RNA polymerase ORF by PCR and inserted into the cloning region of pRB21. This plasmid, containing both the T7 RNA polymerase gene controlled by the early TK promoter and the *lac* repressor gene controlled by an intermediate I1L promoter, was designated pRB21/WX52, and all cloning was carried out in BL21 Star (DE3)pLysS One Shot competent cells (Invitrogen). The Firefly *luc* gene was cloned into pVote.1gfp (46) using an InFusion kit (Clontech) and designated pVote.1gfp/luc.

To make viruses expressing T7 polymerase and LacI, homologous recombination between virus and plasmid or DNA generated by overlapping PCR amplification was carried out as previously described (47) and correct inserts were confirmed by sequencing. Large plaques were picked in BS-C-1 cells and clonally purified. This virus, expressing T7 RNA polymerase and LacI, was designated T7 for simplicity. Recombinant virus T7/luc was made by transfection of T7 virus with pVote.1gfp/luc, and green fluorescence was used to pick and clonally purify plaques. To remove VACV intermediate transcription factor gene A23R, DNA generated by overlapping PCR consisting of (i) 180 bp of the C-terminal end of the A22R ORF, (ii) a dsRed screening marker controlled by the P11 VACV promoter, and (iii) 46 bp of the A24L promoter embedded in the C-terminal end of the A23R ORF and 201 bp of the A24L ORF was inserted into T7/luc to delete the A23R ORF. The resulting virus strain, designated ΔA23T7/luc, was made and propagated in the RK/A8A23 complementing cell line.

Virus ΔA23T7/HA was constructed as follows: the HA ORF from an influenza A virus/PR/8/34 molecular clone was inserted into pVote.1gfp, which was then used for recombination with the ΔA23T7 virus in RK/A8A23 cells. Virus MVA/HA was made as follows: the HA ORF was inserted into the pLW-9 shuttle vector next to the mH5 promoter, which was then transfected into chicken embryo fibroblasts that had been infected with MVA to allow recombination into the deletion III site.

### Single-cycle virus replication and plaque assay.

Single-cycle replication was carried out by infecting RK13, HeLa, and RK/A8A23 cells with 5 PFU of virus per cell. After 2 h of incubation, monolayers were washed twice and fresh medium was added. At 2, 12, 24, and 36 h after infection, cells and medium were harvested from triplicate wells, lysed by three freeze-thaw cycles, and sonicated, and virus titers were determined by plaque assay using RK/A8A23 cells. After 3 days, the cells were immunostained with rabbit anti-VACV antibodies, followed by protein A conjugated with peroxidase (Thermo Fisher Scientific), and incubated with dianisidine substrate until the color developed.

### Antibodies.

The following antibodies were used for immunostaining and Western blotting experiments: polyclonal rabbit anti-VACV (48), anti-T7 RNA polymerase (monoclonal mouse; catalog no. 70566-3, Novagen), anti-LacI (rabbit 600-401-B04S, Rockland), anti-Luc (polyclonal firefly Luc rabbit; catalog no. PA5-32209, Thermo Fisher Scientific), anti-actin (anti-actin rabbit; catalog no. A2066, Sigma-Aldrich), anti-GAPDH (anti-glyceraldehyde-3-phosphate dehydrogenase) (mouse monoclonal clone 1D4; catalog no. MMS-580S, Covance), and mouse anti-influenza virus HA (monoclonal antibody [MAb] H28 E23) (49).

### Western blotting.

Virus-infected cells (3 to 5 PFU/cell) were harvested at 24 h and lysed, and the proteins were resolved by electrophoresis on 4% to 12% NuPage bis-Tris gels and the proteins transferred to a nitrocellulose membrane using an iBlot system (Thermo Fisher). The membranes were blocked with 5% nonfat milk–phosphate-buffered saline (PBS)–0.05% Tween 20 and then incubated overnight at 4°C in the same medium with primary antibodies at appropriate dilutions. The blots were washed to remove excess antibodies, and IRDye 800-conjugated secondary antibodies against mouse or rabbit IgG were added for 1 h at room temperature. After washing was performed, the blots were analyzed and the intensities of the bands were quantified using an Odyssey infrared imager (Li-COR Biosciences).

### Luc expression assay.

Cell monolayers were infected in triplicate with 3 PFU/cell of virus. After 2 h of adsorption at 37°C, the monolayers were washed with PBS and fresh medium was added. At 6, 12, 18, and 24 h after infection, the monolayers were harvested and luminescence was measured according to the instructions of the manufacturer (Promega) using a Sirius model luminometer (Berthold Detection Systems).

### Live bioluminescence imaging.

Mice were inoculated i.m. with 5 × 10^5^ or 5 × 10^6^ PFU of MVA/luc or ΔA23T7/luc. Images were collected after injection of d-luciferine (PerkinElmer) as previously described (50). Uninfected mice served as controls.

A Luc enzyme-linked immunosorbent assay (ELISA) was used to determine Luc antibody titers. Briefly, 96-well plates (Immunlon-2HB; Thermo Fisher Scientific) were coated with 100 ng/well of Firefly Luc (Sigma-Aldrich; catalog no. 9420) diluted in PBS. After overnight incubation at 4°C, ELISA was carried out as previously described (51).

### Influenza virus challenge.

Groups of five 6-to-9-week-old female BALB/c mice (Taconic Biotechnologies) were used. Prior to inoculation, sucrose gradient-purified vectors were diluted in PBS containing 0.05% bovine serum albumin. Virus titers were verified on the day of inoculation by plaque assay. Mice were inoculated with a total of 10^3^, 10^4^, or 10^5^ PFU of virus in 100 µl, with 50 µl injected into the muscle of each hind limb. After 3 weeks, mice were bled via mandibular plexus to obtain sera for antibody determinations. After another week, mice were anesthetized with isoflurane and challenged intranasally with 30 µl of 100 LD_50_ of influenza virus A/PR/8/34. Mice were observed for signs of disease and weighed daily. Mice with a weight loss of ≥30% of their original weight were euthanized in accordance with NIAID Animal Care and Use protocols.

Sera from individual mice were analyzed. Antibodies to influenza HA were determined by a hemagglutination inhibition (HAI) test using 1% turkey red blood cells (Lampire BioLogicals) according to WHO methods (*WHO Animal Influenza Manual on Animal Influenza Diagnosis and Surveillance*, WHO/CDC/CSR/NCS/ 2002.5 E, identification of influenza isolates by hemagglutination inhibition, p 28).

### IFN-α measurements in mice.

Groups of 5 female BALB/c mice, 8 weeks old, were inoculated intravenously with 10^6^ PFU of sucrose gradient-purified MVA/HA virus, ΔA23T7/HA virus, or WR/HA virus in 100 µl of PBS containing 0.05% bovine serum albumin. At 6, 12, and 18 h after infection, mice were bled and sacrificed. Plasma from each mouse was measured for IFN-α levels with a VeriKine mouse IFN-alpha ELISA kit (PBL Assay Science) as described in the manufacturer's instructions.

### Statistical analysis.

The Mann-Whitney test was employed to determine significances of differences in antibody titers in mice by the use of GraphPad Prism version 7.0 (GraphPad Software, Inc.).
